# State-of-the-Art Features for Early-Stage Detection of Diabetic Foot Ulcers Based on Thermograms

**DOI:** 10.3390/biomedicines11123209

**Published:** 2023-12-02

**Authors:** Natalia Arteaga-Marrero, Abián Hernández-Guedes, Jordan Ortega-Rodríguez, Juan Ruiz-Alzola

**Affiliations:** 1 Grupo Tecnología Médica IACTEC, Instituto de Astrofísica de Canarias (IAC), 38205 San Cristóbal de La Laguna, Spain; jortega@iac.es (J.O.-R.); juan.ruiz@ulpgc.es (J.R.-A.); 2 Instituto Universitario de Investigaciones Biomédicas y Sanitarias (IUIBS), Universidad de Las Palmas de Gran Canaria, 35016 Las Palmas de Gran Canaria, Spain; abian.hernandez@ulpgc.es; 3 Instituto Universitario de Microelectrónica Aplicada (IUMA), Universidad de Las Palmas de Gran Canaria, 35017 Las Palmas de Gran Canaria, Spain; 4 Departamento de Señales y Comunicaciones, Universidad de Las Palmas de Gran Canaria, 35016 Las Palmas de Gran Canaria, Spain

**Keywords:** thermography, infrared, deep learning, feature extraction, diabetic foot

## Abstract

Diabetic foot ulcers represent the most frequently recognized and highest risk factor among patients affected by diabetes mellitus. The associated recurrent rate is high, and amputation of the foot or lower limb is often required due to infection. Analysis of infrared thermograms covering the entire plantar aspect of both feet is considered an emerging area of research focused on identifying at an early stage the underlying conditions that sustain skin and tissue damage prior to the onset of superficial wounds. The identification of foot disorders at an early stage using thermography requires establishing a subset of relevant features to reduce decision variability and data misinterpretation and provide a better overall cost–performance for classification. The lack of standardization among thermograms as well as the unbalanced datasets towards diabetic cases hinder the establishment of this suitable subset of features. To date, most studies published are mainly based on the exploitation of the publicly available INAOE dataset, which is composed of thermogram images of healthy and diabetic subjects. However, a recently released dataset, STANDUP, provided data for extending the current state of the art. In this work, an extended and more generalized dataset was employed. A comparison was performed between the more relevant and robust features, previously extracted from the INAOE dataset, with the features extracted from the extended dataset. These features were obtained through state-of-the-art methodologies, including two classical approaches, lasso and random forest, and two variational deep learning-based methods. The extracted features were used as an input to a support vector machine classifier to distinguish between diabetic and healthy subjects. The performance metrics employed confirmed the effectiveness of both the methodology and the state-of-the-art features subsequently extracted. Most importantly, their performance was also demonstrated when considering the generalization achieved through the integration of input datasets. Notably, features associated with the MCA and LPA angiosomes seemed the most relevant.

## 1. Introduction

Diabetic foot ulcers (DFUs) represent the most frequently recognized and highest risk factor associated with diabetes mellitus [1,2]. An infection of the wound may require the amputation of the foot or lower limb. The worldwide estimation is a limb amputation every 20 s [3]. In addition, the recurrence rate remains at about 60% after three years [4]. DFU occurrence can be avoided, reduced, or substantially delayed by early detection, assessment, diagnosis, and tailored treatment [1,5]. The identification of the underlying condition that sustains skin and tissue damage at an early stage, prior to the onset of superficial wounds, is an emerging area of research [6,7,8,9].

Machine learning (ML) and deep learning (DL) approaches based on infrared thermography have been established as a complementary tool for the early identification of superficial tissue damage. Thermography enables real-time visualization of plantar temperature distribution passively, that is, the surface to be measured remains intact [2]. However, the heat pattern of the plantar aspect of the feet and its association with diabetic foot pathologies are subtle and often non-linear [10]. For these reasons, ML and DL models are selected as they offer versatile and highly accurate outputs, lessening the time burden of demanding tasks, the associated costs, and human bias such as subjective interpretations or inherent limitations of human visual perception. Despite the advantages provided, the use of these models as a tool to support clinical decision support systems in real-world scenarios has not been achieved [11]. More studies are required to consider the integration of these models in the healthcare setting [12]. Particularly, in the case of DFUs, the use of ML and DL models is hindered by the lack of labeled data, which causes overfitting and poor generalization on new data if the training dataset is not large enough [13]. There are techniques to mitigate this problem, such as transfer learning [14] or data augmentation [15,16]. Furthermore, these problems are magnified by the current trend towards deeper neural networks [17,18,19], where the problem of vanishing gradients [20] is very widespread; however, skip connections have been proven to work out this limitation and provide other benefits during the training process [21]. Additionally, the lack of standardization regarding feature extraction may also have an impact.

Ideally, ML and DL models should classify subjects at risk of developing an ulcer from a single thermogram containing the plantar aspect of both feet and, if possible, quantify the severity of the lesion. In the context of healthcare, comprehensive data interpretation is crucial. However, in the case of identifying foot disorders using thermography, many features have been proposed in the state of the art, but it is challenging to determine which ones are the most representative for DFUs. The presence of a high number of features can hinder data interpretation. Misinterpretation of the data may lead to inconsistencies among experts when diagnosing a disease, resulting in increased variability in clinical decision-making. Therefore, the identification of foot disorders using thermography requires establishing a subset of relevant features to reduce decision variability and data misinterpretation and provide a better overall cost–performance for classification [22]. Using a subset of features with relevant information, classifiers with better cost–performance ratios are achieved, as reducing the number of features can lessen both computational and memory resources [23]. The lack of standardization among thermograms as well as the unbalanced datasets towards diabetic cases hinder the establishment of this suitable subset of features.

ML and DL models have been explored to determine relevant features for early detection of DFUs [9,24,25,26,27]. However, except for a few cases, these studies were derived mainly from the only publicly available dataset, the INAOE dataset (Instituto Nacional de Astrofísica, Óptica y Electrónica) [26], which is composed of thermograms containing the plantar aspect of both feet. Recently, a similar dataset was released, STANDUP [28], which provides means for extending the current state of the art by simply increasing the number of samples available to train the ML and DL models. Furthermore, the additional dataset enables the determination of the generalizability of the set of state-of-the-art features previously extracted by classical and DL approaches [27].

In this work, the same methodology previously described was executed in order to extract a state-of-the-art set of features from infrared thermograms [27]. Four input datasets were considered by merging different datasets for feature extraction. A subset of features associated with each input dataset was extracted using classical- and DL-based approaches. The subset of features common to all of the approaches employed were used as an input for both a standard and an optimized support vector machine (SVM) [29] classifier. The SVM classifier was used as a reference to assess and compare the performance of each set of extracted features from the STANDUP and extended databases. In addition, a comparison was performed between the more relevant and robust features extracted in this work and those extracted using solely the INAOE dataset [27] as well as those proposed in previous studies [9].

## 2. Materials and Methods

### 2.1. Datasets Description

The datasets employed throughout this work are composed of infrared (IR) images acquired with different sensors, ambient conditions, and sites. RGB images of the same scene are also available for all datasets. These images are usually used to aid in the segmentation of the feet sole from the background [30]. In addition, the corresponding acquisition campaigns were carried out at separate time points over a different population sample. Further details regarding the infrared sensors and the protocol employed for image acquisition can be found elsewhere for the INAOE [26], STANDUP [28], and local datasets [30,31].

#### 2.1.1. STANDUP Dataset

The STANDUP dataset, released in June 2023, was generated for DFU detection based on infrared thermography [28]. The dataset is composed of samples from 227 subjects, 145 diabetic and 82 healthy ones. For each subject, images were acquired at T0 after 10 min in a resting position to reach a state of thermodynamic equilibrium and at T10 after a cold stress test. The diabetic group was divided into three subgroups: R0 corresponded to non-neuropathic and non-ischemic individuals; R1 included neuropathic individuals without ischemia; and R2 was composed of individuals with ischemia. No distinction was made regarding the grading of the pathology in the present work; that is, diabetic and healthy subjects were considered for classification. Furthermore, the analysis is primarily focused on images acquired at T0, facilitating meaningful comparisons with other existing datasets (such as INAOE and local). That is, thermograms corresponding to subjects who underwent a cold stress test were not considered. Thus, the STANDUP dataset was reduced to 145 diabetic and 38 healthy subjects. Notice that, similarly to that observed for the INAOE dataset, this dataset is strongly unbalanced toward diabetic cases.

#### 2.1.2. INAOE Dataset

The INAOE thermogram dataset, released in December 2019, is composed of samples from 167 volunteers, 122 diabetic and 45 non-diabetic subjects [26]. Notice that the INAOE dataset is unbalanced toward diabetic cases. This dataset was originally intended to study the distribution of temperature in the plantar region among diabetic and non-diabetic subjects. However, these thermograms have been widely used for DFU detection at an early stage [9,27,32,33].

#### 2.1.3. Local Dataset

The local dataset was acquired in 2021 [30,31] and is being publicly released in association with the present work. This dataset contains samples from 22 healthy volunteers acquired at four different time points, although only the images corresponding to the 15 min resting position prior to acquisition were employed. This dataset was merged into the INAOE dataset, providing an extended dataset aiming to compensate the imbalance toward diabetic cases exhibited by the INAOE dataset.

### 2.2. Feature Extraction

The features were extracted from the INAOE and local datasets as previously described [27] and following the workflow initially proposed for the INAOE dataset [25,26]. However, for the STANDUP dataset, some preprocessing was required. First, as mentioned above, only images at T0 were employed. The thermograms were provided as grayscale images without temperature values, which prevented the extraction of certain features. For this reason, the color bar within each infrared image was used to define the highest and lowest temperature. Thus, the grayscale values were converted to temperature values. The infrared images were then segmented using the Segment Anything Model (SAM) [34]. In order to extract the angiosomes, a composite unit of tissues supplied by an artery [25,26], the segmented images were split to process each foot separately. By considering these angiosomes, the foot was divided into four regions: medial plantar artery (MPA), lateral plantar artery (LPA), medial calcaneal artery (MCA), and lateral calcaneal artery (LCA). As previously set, a temperature threshold of 18 °C was employed as the lower limit. This caused the average values for certain angiosomes to be zero. Therefore, only subjects for which all angiosomes were not null in both feet were further considered. Overall, the dataset was reduced to 88 diabetic and 34 healthy subjects.

The nomenclature employed to name the extracted features mentioned above consisted of using a letter to specify the foot, ‘L’ for left and ‘R’ for right, followed by the name of the corresponding angiosome. For the features extracted using the entire foot, this second descriptor was discarded. Then, the variable was set using lowercase letters such as mean, std, max, min, skew, or kurtosis. Capital letters were employed for the thermal change index (TCI), hot spot estimator (HSE), estimated temperature (ET), and estimated temperature difference (ETD) as well as for normalized temperature ranges (NTRs) followed by the subsequent class.

Four sets of features were extracted in this work depending on the input dataset. The first set, henceforth named DFU, was composed of the features extracted using the INAOE and local datasets. The second contained features solely for the STANDUP dataset, defined as STANDUP. In addition, the STANDUP dataset was merged with the local dataset. The set of associated features was named as STANDUP${}_{2}$. The final set of features, defined as ALL, was extracted by merging all datasets: INAOE, local, and STANDUP. The distribution between diabetic and healthy subjects for each dataset were 88/34, 88/56, 210/101 for the STANDUP, STANDUP${}_{2}$, and ALL datasets, respectively. As previously mentioned [27], the input datasets, composed by the features extracted from thermograms, were modified to compensate for the imbalance between classes using SMOTE (Synthetic Minority Over-sampling TEchnique), which generates new samples by linear interpolation between samples from the minority class. That is, prior to the execution of the workflow, the input datasets are balanced by generating samples composed of features for the healthy subjects. Therefore, 88 sets of features of thermograms for each class were available for the STANDUP and STANDUP${}_{2}$ datasets, and 210 were available for the ALL dataset.

#### 2.2.1. Feature Selection

The number of features or input variables per dataset was 188 and, as previously reported, a detailed investigation was proposed to detect the most relevant features [27]. The approaches employed included some classical methods, random forest and lasso, as well as two innovative ones based on DL, named concrete and variational dropout.

For the DL approaches, two methods for feature selection have been explored based on the variational inference in the input layer. The first, based on the concrete dropout approach [35], approximates a feature selection mechanism with a $L_{0}$ regularization factor using a Bernoulli distribution [36]. The second approach, called variational dropout, incorporates Gaussian noise with variational parameters that are learned during training. This method has been demonstrated to produce sparse representations [37] and, for this reason, it has been proposed as a feature selection technique [27].

Initially, the original input set was optimized by removing highly correlated variables using the Pearson correlation coefficient [38]. Those features with a correlation of r>0.95 were considered highly correlated and, therefore, a reduction in the number of features was performed. The number of features or input variables was reduced from 188 to 141 for the DFU and STANDUP${}_{2}$ datasets, while for the STANDUP and ALL datasets, the final number of features was 139 and 144, respectively. Subsequently, feature ranking based on logistic regression and an AUC-ROC (area under the curve of the receiver operating characteristic) analysis [39] were proposed to select the most informative features.

Five-fold cross-validation was employed and, for each iteration, the dataset was split into training and testing using a 80:20 ratio, respectively. Therefore, the relevance of the features was established as the average value resulting from the five iterations. Furthermore, as previously indicated, a batch size of 32 samples, 500 training epochs, and an ADAM optimizer [40] were employed during the training process of the DL-based models. The parameters $\beta_{1}$ and $\beta_{2}$, which control the exponential decay rates for the moment estimation, were set to 0.9 and 0.999, respectively. The learning rate (lr) was set to $10^{-2}$ for the concrete variational feature selector and $10^{-3}$ for the variational dropout [27].

#### 2.2.2. Classification

**Figure 1:**
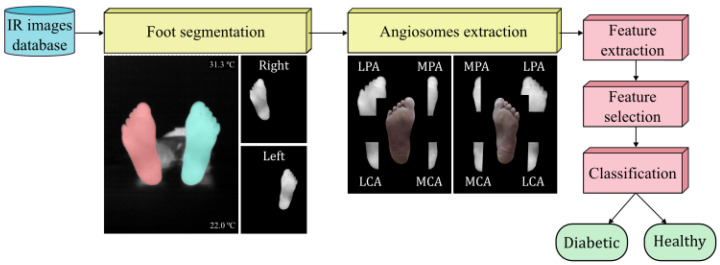
Flowchart representing the experimental procedure.

The objective is to identify a set of relevant features to classify the thermograms into diabetic and non-diabetic. An SVM [29] classifier was used with each dataset as an input to quantify the performance of the extracted features, their rank, and selected combination. The different steps of the entire workflow are illustrated in Figure 1.

Two settings were considered. In the first case, the SVM classifier was not optimized, and standard hyperparameters were chosen to offer a fair comparison between the proposed approaches to rank the features. In the second case, the SVM classifier was optimized using the randomized search [41] to obtain the best parameters for each set of features. The main hyperparameters associated with the SVM classifier were γ and *C*. A Gaussian kernel, also known as the radial basis function (RBF) kernel, has a hyperparameter, γ, which controls the spread of the Gaussian center. The hyperparameter *C*, used to direct the penalty $L_{2}$, controls the trade-off between decision boundary and misclassification.

## 3. Results

### 3.1. Selected Features

Following the workflow described [27], features were ranked for each approach: lasso, random forest, concrete, and variational dropout. The 10 first features extracted for each approach were considered the most relevant and were fed to the optimized and non-optimized SVM classifier. The non-optimized SVM hyperparameters were established as 0.1 and 1 for γ and *C*, respectively, using an RBF kernel. For informative purposes, the first 10 features extracted from each approach using the standard SVM classifier are listed in Appendix A.

Notice that three sets of features were employed as inputs: STANDUP, STANDUP${}_{2}$, and ALL. Therefore, the respective SVM hyperparameters varied according to the input dataset. In all cases, the best model was found using an RBF kernel. The values of the hyperparameter γ were 0.004, 0.002, and 0.007, whereas *C* values were 26.827, 51.795, and 6.551 for the STANDUP, STANDUP${}_{2}$, and ALL datasets, respectively.

The features that consistently appeared in all implemented approaches are listed in Table 1, organized by their respective ranks and datasets. The ranks of these features changed according to the approach employed; thus, the lowest rank among the different approaches was assigned as its final rank. Notice that only features found up to a rank of lower than 50 were considered. Additionally, the 10 best-ranked features selected by the different feature selection methods for each dataset can be found in Appendix A.

As can be observed, the number of features in coincidence varied depending on the input dataset. R_MCA_std and R_LCA_kurtosis appeared as relevant features independent of the approach and dataset (highlighted in Table 1). R_MCA_kurtosis and LPA_ETD appeared in coincidence for three datasets. In addition, nine features were in coincidence for two datasets: L_kurtosis, R_kurtosis, L_MPA_kurtosis, L_MCA_std, R_LPA_std, R_MPA_HSE, L_min, L_LCA_std, and L_LPA_min. In summary, 13 features appeared as relevant in at least 2 different datasets, with 3 of them corresponding to the entire foot and the rest being distributed within the angiosomes. Three features were associated with the MCA and LPA angiosomes, respectively, where two features were linked to the MPA and LCA angiosomes.

### 3.2. Classification Using a Standard SVM

To prevent bias in the conclusion, a standard hyperparameter configuration was employed for the SVM classifier. This approach assumed that the results were not inflated due to an overfitting hyperparameter setting tailored to the selected features.

The top 10 ranked features from each approach and dataset (see Appendix A) were used to train the standard SVM classifier, that is, the SVM with a fixed hyperparameter configuration. Furthermore, the features that consistently ranked in the top 10 across all approaches, as depicted in Table 1, were also used as input features.

In order to facilitate comparison with previous studies, two sets of features were considered. The first set was composed of the following features [27]: R_LPA_min, R_MCA_std, Foot_ETD, LPA_ETD, L_MCA_std, L_kurtosis, L_LPA_std, R_kurtosis, R_LCA_std, and R_LCA_kurtosis, which corresponded to the top 10 features presented in Table 1 (first column). The second set was composed of the following ten ranked features [9]: TCI, NTR_C${}_{4}$, NTR_C${}_{3}$, MPA_mean, LPA_mean, LPA_ET, LCA_mean, highest temperature, NTR_C${}_{2}$, and NTR_C${}_{1}$.

The results for the standard SVM classifier for all approaches employed are listed in Table 2, Table 3 and Table 4 for the STANDUP, STANDUP${}_{2}$, and ALL datasets, respectively.

The performance metrics were significantly reduced when the input dataset presented higher heterogeneity, as occurred when merging datasets. This is the case for the STANDUP${}_{2}$ and ALL datasets. Regarding the best approach, for the STANDUP dataset, the highest accuracy and F1-score were observed for the lasso approach, although very close values were found for the variational dropout approach. The highest precision was noticed for the variational dropout approach, whereas the best recall was shown for the workflow described in this work to extract state-of-the-art features. For the STANDUP${}_{2}$ dataset, the highest accuracy and precision were found for the random forest and lasso approaches, respectively. Recall and F1-score were best for the concrete dropout approach. Finally, for the ALL dataset, the highest metrics were observed for state-of-the-art features extracted from a previous work [27].

### 3.3. Classification Using an Optimized SVM

In this section, the classification metrics results were depicted by using the well-fitted hyperparameters setting per dataset. Similarly to the previous section, the results for the optimized SVM using all approaches are shown in Table 5, Table 6 and Table 7 for the STANDUP, STANDUP${}_{2}$, and ALL datasets, respectively.

A consistent trend was observed with the optimized SVM, mirroring the findings for the standard SVM. When the dataset’s heterogeneity increased, the performance metrics decreased. In terms of the best-performing approach, for the STANDUP dataset, the highest accuracy, precision, and F1-score were observed in the lasso approach. However, the best recall was found for the state-of-the-art features extracted from a previous work [27]. For the STANDUP${}_{2}$ dataset, the highest accuracy, recall, and F1-score were found in the random forest approach, whereas the lasso approach provided the best precision. For the ALL dataset, the best performance metrics were observed in the lasso approach, except for the recall, which was best in the variational dropout approach.

### 3.4. Effects of Class Balance by SMOTE

**Figure 2:**
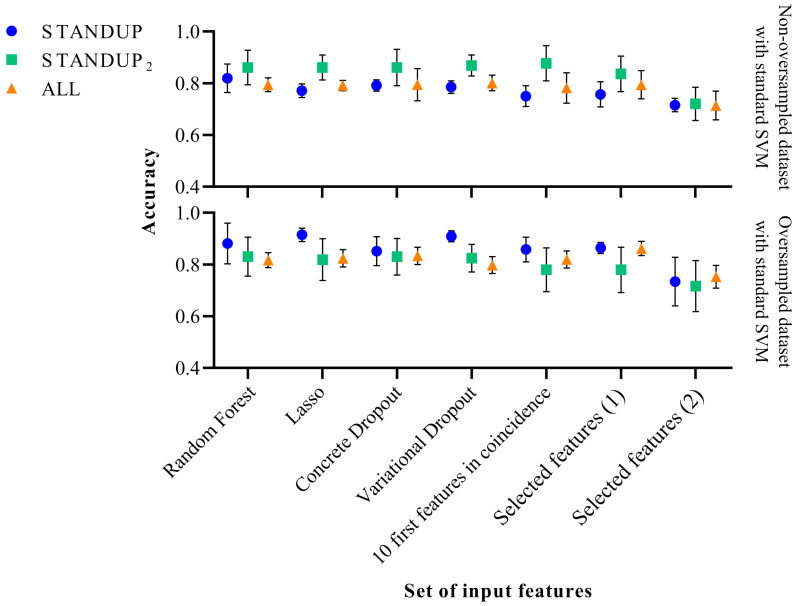
Performance comparison of the standard SVM classifier before (**above**) and after SMOTE (**below**). Selected features (1) [27] and Selected features (2) [9] refer to a subset of features extracted in previous publications.

**Figure 3:**
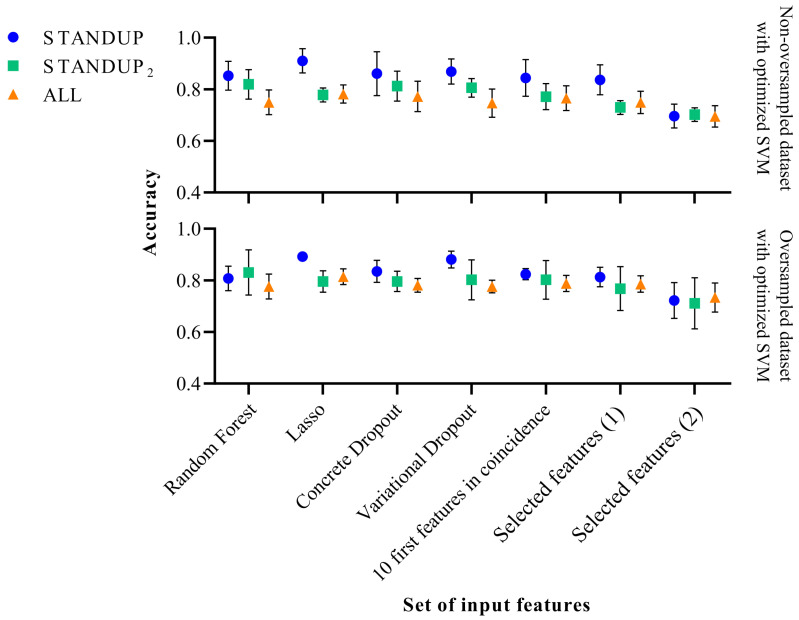
Performance comparison of the optimized SVM classifier before (**above**) and after SMOTE (**below**). Selected features (1) [27] and Selected features (2) [9] refer to a subset of features extracted in previous publications.

As mentioned above, SMOTE was used to compensate for the imbalance between the classes of the respective datasets. However, the effects of this procedure were not quantified. Therefore, the workflow illustrated in Figure 1 was additionally executed without the oversampling step to compare the modifications observed on the performance metrics of the respective approaches and datasets. The results are shown in Figure 2 and Figure 3 when applying the standard and optimized SVM classifier, respectively.

In the case of applying the SVM classifier with the standard hyperparameters, a slight improvement in classification performance was observed with the STANDUP dataset with oversampling. However, it decreased moderately when oversampling the STANDUP${}_{2}$ dataset. Moreover, while considering the ALL dataset, the more heterogeneous one, the performance remained similar, as shown in Figure 2. In general, the variability of the different approaches increases when using the non-oversampled ALL dataset, whereas the opposite trend is observed for the other datasets.

On the other hand, the accuracy displayed when applying the optimized SVM decreased for the STANDUP dataset, as can be observed from Figure 3. For the STANDUP${}_{2}$ dataset, the performance accuracy was maintained. Finally, for the ALL dataset, the accuracy increased when using the oversampling. Additionally, the variability decreased for the oversampled STANDUP and ALL datasets, whereas it increased for the STANDUP${}_{2}$ dataset. Furthermore, a steeper decrement in performance was noticed for the non-oversampled dataset, particularly for the selected features.

It is worth noting that the features previously proposed [9] tend to have the highest variability among the different datasets. Moreover, the STANDUP dataset using the lasso approach for feature selection has the highest accuracy after applying SMOTE (see Figure 2 and Figure 3). This may be a consequence of the linear approach used for class balance by the SMOTE method. Nevertheless, the features selected by the variational dropout approach provided close performance metrics. However, the features selected by the variational dropout approach were consistent regarding the variability among the different settings.

## 4. Discussion

Several approaches were considered to extract relevant features used for DFU detection based on infrared thermograms following the same methodology previously described [27]. In this case, an extended and multicenter dataset was created by merging the INAOE, STANDUP, and local database, which provided a generalization factor to the classification task at hand. This was conducted to determine whether a thermogram corresponded to a healthy or diabetic person.

To the best of the authors’ knowledge, this is the largest thermogram dataset explored, especially regarding DFU detection at an early stage. As mentioned above, the INAOE dataset has been the only thermogram database publicly available, and the recently released STANDUP dataset provides the opportunity to test the methodology previously established. The STANDUP dataset was considered alone as well as merged with the local dataset aiming to correct the imbalance toward diabetic cases observed. Furthermore, a more generalized and extended dataset was created by merging all available datasets (ALL).

Classical approaches, such as lasso and random forest, were tested against two DL-based approaches by applying the dropout techniques, concrete and variational dropout. The dropout techniques, initially designed to address overfitting in DL models, were employed not only in the feature selection but also across different layers using a dropout rate of 0.5. For instance, in the case of concrete dropout, the input layer is defined by variational parameters establishing a binomial distribution composed of *d* independent Bernoulli ‘continuous relaxed’ distributions [27]. This configuration acts as a ‘gate’ to identify irrelevant features by introducing noise [27]. In an ideal scenario, relevant features tend to have a dropout rate of close to zero, while irrelevant features tend towards a dropout rate of one. In essence, the proposed restriction in the model implicitly serves to mitigate overfitting concerns inherent in DL-based models. Furthermore, it is worth noting that the chosen models, particularly the random forest and DL-based approaches, are inherently robust at handling data variability. While preprocessing could mitigate issues related to feature extraction, the focus of this work was to identify the most relevant features within the newly released STANDUP and ALL databases and compare them with previous results [27]. Therefore, extensive hard preprocessing of the thermograms was avoided.

In the context of ML models, where the parameters are denoted as θ, theoretically, a test could be established to validate the statistical significance of p(X,Y|θ) concerning p(θ), where *X* is the dataset and *Y* the prediction. However, it is crucial to note that ML models are commonly evaluated using metrics such as the mean squared error (MSE) or the AUC-ROC. In this work, K-fold cross-validation [42] was employed to validate the SVM model. The dataset is partitioned into ‘k’ subsets, and the model is trained on ‘k − 1’ subsets while being validated on the remaining subset. This process is iterated ‘k’ times, with each ‘fold’ serving as both a training and test set. The outcome is an estimation of the mean error value and standard deviation, providing a robust assessment of model performance. Specifically, a low standard deviation was observed for the standard SVM classifier with predefined hyperparameter configurations across different experiments to discard biased conclusions. This finding leads to the conclusion that the model effectively fits the distribution p(X,Y) and the provided features contain sufficient information about *X* for predicting *Y*. In general, the uncertainty is increased with the class-balanced dataset, as noticed by the increase in the standard deviation.

The analysis of the subset of features considered relevant and the subsequent classification task for each approach provided sufficient metric values regarding performance. For the dataset with maximum heterogeneity (ALL), the best approach varied depending on whether the classifier was standard or optimized. For the standard SVM, in which a true comparison can be drawn between the different approaches, the best performance metrics were observed for the state-of-the-art features previously reported [27]. These results support the fact that the methodology, and the subset of state-of-the-art features subsequently derived, provide consistent and reliable descriptors to discriminate between healthy and diabetic individuals. Despite the heterogeneity of the dataset, the performance was suitable, although some decreases were observed precisely due to this variability. The best F1-score reported for the DFU dataset was 0.9027 ± 0.104 [27], whereas the same metrics was 0.8513 ± 0.0279 for the ALL dataset.

For the optimized SVM, the lasso approach provided the best performance metrics, except for the recall, which was best in the variational dropout approach. In this case, the F1-score for the ALL dataset was 0.7956 ± 0.0291. The reason for a decrement in the performance may be due to oversampling. For the non-oversampled datasets, when using the optimized SVM, the recall performance increases. This can be due to the fact that some subjects considered as control may be diabetic. Therefore, when applying SMOTE, features corresponding to diabetic subjects are propagated and disrupt the control group. This is particularly noticed for the STANDUP dataset.

Regarding the set of relevant features, R_MCA_std and R_LCA_kurtosis appeared as relevant features independent of the approach and dataset. LPA_ETD appeared in coincidence for three datasets:,DFU, STANDUP, and STANDUP${}_{2}$, whereas R_MCA_kurtosis also appeared in coincidence for three datasets, STANDUP, STANDUP${}_{2}$, and ALL. In addition, nine features were in coincidence for two datasets. Among all of these features found in coincidence, those that already appeared as relevant in our previous work are as follows [27]: R_MCA_std, LPA_ETD, L_kurtosis, R_kurtosis, L_MCA_std, and R_LPA_std. Thus, these features, mainly associated with the MCA and LPA angiosomes, as well as the kurtosis for each foot, consistently appeared as relevant features independent of the input dataset.

A major limitation of the present study is the lack of an associated clinical trial. At this stage, the main aim was focused on establishing the workflow required for data analysis. In this work, as a proof of concept, a relevant set of state-of-the-art features was determined. This provided a tool to successfully discriminate between healthy and pathological subjects by measuring the temperature within the plantar aspects of both feet. Furthermore, some insight was gained regarding the importance of the different angiosomes and their predictive value for classification. However, the presented methodology must be tested and validated in a standard clinical setting in order to assess the clinical relevance of the findings. Then, the incorporation of glycemic control parameters and other diabetes-specific factors must be included as additional features. This allows for the assessment of whether underlying biochemical processes relate to inflammation or microvascular changes in diabetic foot disorders.

Further studies would require more balanced datasets to classify thermograms between two classes, diabetic and healthy. The necessity of additional preprocessing to unify different datasets must be explored. The lack of improvement noticed in this work when merging datasets in comparison with our previous work [27] may be a consequence of avoiding a uniform preprocessing.

Moreover, the STANDUP database provides thermographic images after thermal stress for healthy and diabetic subjects. This could help to gain some insight regarding dynamic thermal changes in diabetic foot disorders and whether thermal information could contribute to early detection. Currently, these data are being preprocessed in order to apply the methodology presented in this work. Finally, once the patient has been labeled as diabetic, a new classification task is planned to determine the level of severity within diabetic thermograms.

## 5. Conclusions

The identification of foot disorders at an early stage using thermography requires establishing a subset of relevant features to reduce decision variability and data misinterpretation and provide an overall better cost–performance for classification. The lack of standardization among thermograms as well as the unbalanced datasets towards diabetic cases hinder the establishment of this suitable subset of features. In this work, an extended and more generalized dataset has been employed. The suitability of the methodology employed has been confirmed and, most importantly, the performance of the state-of-the-art features previously proposed was demonstrated, despite the generalization added by the merged input datasets. Finally, features associated with the MCA and LPA angiosomes seemed the most relevant.

## Figures and Tables

**Table 1:** Most relevant features that coincide in all approaches considered, listed according to rank and input dataset. Features that coincide among all datasets were highlighted.

Rank	Features in Coincidence (DFU) [27]	Features in Coincidence (STANDUP)	Features in Coincidence (STANDUP2)	Features in Coincidence (ALL)
**Rank < 10 **	R_LPA_min	**R_MCA_std**	**R_MCA_std**	**R_MCA_std**
		R_MPA_kurtosis		
**Rank < 20**	**R_MCA_std**	R_MCA_kurtosis	R_MCA_kurtosis	L_LPA_min
		L_kurtosis	L_MCA_min	L_MPA_std
		R_kurtosis	L_LPA_min	R_MCA_kurtosis
**Rank < 30**	Foot_ETD	L_min	L_LCA_std	**R_LCA_kurtosis**
	LPA_ETD	R_MPA_skew	L_MPA_kurtosis	
	L_MCA_std	**R_LCA_kurtosis**		
	L_kurtosis			
**Rank < 50**	L_LPA_std	L_LCA_std	**R_LCA_kurtosis**	L_MCA_std
	R_kurtosis	L_MPA_kurtosis	R_MPA_std	R_MCA_skew
	R_LCA_std	R_LCA_skew	R_max	L_MPA_HSE
	**R_LCA_kurtosis**	L_MPA_min	R_LCA_min	R_LPA_std
	R_LPA_std	L_MCA_kurtosis	L_LCA_min	R_LPA_skew
	R_MPA_HSE	R_LPA_HSE	L_MPA_skew	R_MPA_HSE
	LCA_ETD	LPA_ETD	LPA_ETD	
			R_MCA_NRT_C${}_{4}$	
			L_min	
			L_MCA_skew	

**Table 2:** Performance metrics of the approaches considered for the STANDUP dataset and the standard SVM classifier according to the selected input features shown in Table 1. The highest value for each performance metric is highlighted.

Input (STANDUP)	Approach	Accuracy	Precision	Recall	F1-Score
10 first features	**Lasso**	**0.9146 ± 0.0259**	0.9608 ± 0.0330	0.8604 ± 0.0524	**0.9070 ± 0.0357**
	**Random Forest**	0.8811 ± 0.0785	0.8998 ± 0.0771	0.8670 ± 0.0914	0.8815 ± 0.0777
	**Concrete Dropout**	0.8518 ± 0.0559	0.8386 ± 0.0716	0.8697 ± 0.0877	0.8509 ± 0.0608
	**Variational Dropout**	0.9091 ± 0.0215	**0.9889 ± 0.0222**	0.8298 ± 0.0479	0.9011 ± 0.0231
10 first features in coincidence	**Lasso, Random Forest, Concrete, and Variational Dropout**	0.8579 ± 0.0478	0.8265 ± 0.0464	**0.9121 ± 0.0442**	0.8668 ± 0.0419
Selected features from [27]	**Lasso, Random Forest, Concrete, and Variational Dropout**	0.8637 ± 0.0212	0.8480 ± 0.0193	0.8883 ± 0.0291	0.8672 ± 0.0138
Selected features from [9]	**Pearson, Chi-square, RFE, Logistics, Random Forest, and LightGBM**	0.7337 ± 0.0937	0.7963 ± 0.1308	0.6552 ± 0.1286	0.7076 ± 0.1040

**Table 3:** Performance metrics of the approaches considered for the STANDUP${}_{2}$ dataset and the standard SVM classifier according to the selected input features shown in Table 1. The highest value for each performance metric is highlighted.

Input (STANDUP${}_{2}$)	Approach	Accuracy	Precision	Recall	F1-Score
10 first features	**Lasso**	0.8186 ± 0.0808	**0.8302 ± 0.0634**	0.8125 ± 0.1309	0.8163 ± 0.0840
	**Random Forest**	**0.8303 ± 0.0756**	0.8150 ± 0.0886	0.8653 ± 0.0743	0.8363 ± 0.0662
	**Concrete Dropout**	0.8300 ± 0.0706	0.7980 ± 0.0418	**0.8929 ± 0.0932**	**0.8414 ± 0.0611**
	**Variational Dropout**	0.8243 ± 0.0534	0.8062 ± 0.0494	0.8672 ± 0.0676	0.8331 ± 0.0405
10 first features in coincidence	**Lasso, Random Forest, Concrete, and Variational Dropout**	0.7794 ± 0.0849	0.7996 ± 0.1186	0.7876 ± 0.1008	0.7834 ± 0.0620
Selected features from [27]	**Lasso, Random Forest, Concrete, and Variational Dropout**	0.7792 ± 0.0877	0.8256 ± 0.1233	0.7552 ± 0.1192	0.7762 ± 0.0727
Selected features from [9]	**Pearson, Chi-square, RFE, Logistics, Random Forest, and LightGBM**	0.7164 ± 0.0989	0.7216 ± 0.1157	0.7028 ± 0.1349	0.7064 ± 0.1161

**Table 4:** Performance metrics of the approaches considered for the ALL dataset and the standard SVM classifier according to the selected input features shown in Table 1. The highest value for each performance metric is highlighted.

Input (ALL)	Approach	Accuracy	Precision	Recall	F1-Score
10 first features	**Lasso**	0.8238 ± 0.0332	0.8629 ± 0.0465	0.7740 ± 0.0479	0.8144 ± 0.0315
	**Random Forest**	0.8167 ± 0.0342	0.8931 ± 0.0167	0.7219 ± 0.0520	0.7973 ± 0.0331
	**Concrete Dropout**	0.8333 ± 0.0337	0.8826 ± 0.0432	0.7737 ± 0.0785	0.8211 ± 0.0389
	**Variational Dropout**	0.7976 ± 0.0328	0.7906 ± 0.0582	0.8161 ± 0.0367	0.8012 ± 0.0288
10 first features in coincidence	**Lasso, Random Forest, Concrete, and Variational Dropout**	0.8191 ± 0.0332	0.8873 ± 0.0608	0.7332 ± 0.0726	0.7997 ± 0.0458
Selected features from [27]	**Lasso, Random Forest, Concrete, and Variational Dropout**	**0.8619 ± 0.0278**	**0.9250 ± 0.0330**	**0.7931 ± 0.0647**	**0.8513 ± 0.0279**
Selected features from [9]	**Pearson, Chi-square, RFE, Logistics, Random Forest, and LightGBM**	0.7524 ± 0.0442	0.8220 ± 0.0665	0.6554 ± 0.0454	0.7268 ± 0.0355

**Table 5:** Performance metrics of the approaches considered for the STANDUP dataset and the optimized SVM classifier according to the selected input features shown in Table 1. The highest value for each performance metric is highlighted.

Input (STANDUP)	Approach	Accuracy	Precision	Recall	F1-Score
10 first features	**Lasso**	**0.8921 ± 0.0112**	**0.9615 ± 0.0317**	0.8193 ± 0.0370	**0.8834 ± 0.0099**
	**Random Forest**	0.8071 ± 0.0474	0.8009 ± 0.0363	0.8233 ± 0.0799	0.8097 ± 0.0465
	**Concrete Dropout**	0.8348 ± 0.0429	0.8661 ± 0.0622	0.7927 ± 0.0630	0.8256 ± 0.0476
	**Variational Dropout**	0.8808 ± 0.0328	0.9599 ± 0.0329	0.7982 ± 0.0467	0.8706 ± 0.0302
10 first features in coincidence	**Lasso, Random Forest, Concrete, and Variational Dropout**	0.8238 ± 0.0217	0.8370 ± 0.0319	0.8088 ± 0.0501	0.8208 ± 0.0177
Selected features from [27]	**Lasso, Random Forest, Concrete, and Variational Dropout**	0.8127 ± 0.0375	0.8060 ± 0.0368	**0.8304 ± 0.0297**	0.8174 ± 0.0259
Selected features from [9]	**Pearson, Chi-square, RFE, Logistics, Random Forest, and LightGBM**	0.7219 ± 0.0693	0.7799 ± 0.0734	0.6460 ± 0.1357	0.6952 ± 0.0766

**Table 6:** Performance metrics of the approaches considered for the STANDUP${}_{2}$ dataset and the optimized SVM classifier according to the selected input features shown in Table 1. The highest value for each performance metric is highlighted.

Input (STANDUP${}_{2}$)	Approach	Accuracy	Precision	Recall	F1-Score
10 first features	**Lasso**	0.7956 ± 0.0414	**0.8244 ± 0.0790**	0.7725 ± 0.0795	0.7909 ± 0.0342
	**Random Forest**	**0.8303 ± 0.0876**	0.7996 ± 0.0915	**0.8892 ± 0.0940**	**0.8396 ± 0.0803**
	**Concrete Dropout**	0.7959 ± 0.0340	0.7983 ± 0.0668	0.8087 ± 0.0494	0.7995 ± 0.0194
	**Variational Dropout**	0.8019 ± 0.0775	0.7958 ± 0.0645	0.8245 ± 0.1035	0.8071 ± 0.0695
10 first features in coincidence	**Lasso, Random Forest, Concrete, and Variational Dropout**	0.8019 ± 0.0753	0.8012 ± 0.0842	0.8232 ± 0.0950	0.8074 ± 0.0620
Selected features from [27]	**Lasso, Random Forest, Concrete, and Variational Dropout**	0.7679 ± 0.0852	0.8152 ± 0.1170	0.7094 ± 0.0782	0.7549 ± 0.0795
Selected features from [9]	**Pearson, Chi-square, RFE, Logistics, Random Forest, and LightGBM**	0.7108 ± 0.0990	0.7381 ± 0.1423	0.6836 ± 0.0888	0.7030 ± 0.0967

**Table 7:** Performance metrics of the approaches considered for the ALL dataset and the optimized SVM classifier according to the selected input features shown in Table 1. The highest value for each performance metric is highlighted.

Input (ALL)	Approach	Accuracy	Precision	Recall	F1-Score
10 first features	**Lasso**	**0.8143 ± 0.0307**	**0.8937 ± 0.0592**	0.7259 ± 0.0773	**0.7956 ± 0.0291**
	**Random Forest**	0.7762 ± 0.0485	0.8095 ± 0.0825	0.7374 ± 0.0562	0.7681 ± 0.0409
	**Concrete Dropout**	0.7810 ± 0.0267	0.8582 ± 0.0648	0.6852 ± 0.0773	0.7558 ± 0.0333
	**Variational Dropout**	0.7762 ± 0.0243	0.7891 ± 0.0595	**0.7586 ± 0.0247**	0.7718 ± 0.0245
10 first features in coincidence	**Lasso, Random Forest, Concrete, and Variational Dropout**	0.7881 ± 0.0314	0.8447 ± 0.0693	0.7118 ± 0.0404	0.7702 ± 0.0324
Selected features from [27]	**Lasso, Random Forest, Concrete, and Variational Dropout**	0.7857 ± 0.0319	0.8736 ± 0.0631	0.6796 ± 0.0802	0.7582 ± 0.0401
Selected features from [9]	**Pearson, Chi-square, RFE, Logistics, Random Forest, and LightGBM**	0.7333 ± 0.0561	0.8044 ± 0.0705	0.6338 ± 0.0764	0.7039 ± 0.0515

## Data Availability

The INAOE [26] and STANDUP [28] datasets employed in this study are publicly available at https://ieee-dataport.org/open-access/plantar-thermogram-database-study-diabetic-foot-complications (accessed on 27 November 2023) and https://www.standupproject.eu/manager/?conf=default&route=/STANDUP_Database (accessed on 27 November 2023), respectively. The local dataset has been made available at https://www.iac.es/en/science-and-technology/technology-transfer-iactec/forms (accessed on 27 November 2023). Notice that, due to privacy restrictions, only 19 of the 22 image sets originally acquired were released. The code implemented for this work is freely available at https://github.com/mt4sd/DFUFeatureRankingByVariationalDropout (accessed on 27 November 2023).

## Appendix A

The first 10 features extracted for each employed approach are listed according to the input dataset.

**Table A1:** The 10 most relevant features extracted from the STANDUP dataset, listed according to rank for all approaches considered: lasso, random forest, concrete, and variational dropout.

Rank	Lasso	Random Forest	Concrete Dropout	Variational Dropout
1	R_MPA_kurtosis	R_std	L_min	L_LPA_min
2	R_MCA_std	R_MCA_std	R_MPA_skew	R_MCA_std
3	R_max	R_MPA_std	R_LCA_kurtosis	R_MPA_skew
4	R_MPA_skew	R_MPA_kurtosis	R_MPA_kurtosis	R_MCA_kurtosis
5	R_LPA_NRT_C${}_{7}$	L_LCA_std	L_LPA_min	R_MPA_kurtosis
6	R_MPA_NRT_C${}_{4}$	L_MPA_std	R_MCA_kurtosis	R_LPA_NRT_C${}_{7}$
7	R_LCA_kurtosis	L_std	R_MCA_std	L_min
8	R_MCA_kurtosis	L_kurtosis	L_kurtosis	R_LPA_NRT_C${}_{2}$
9	R_MCA_NRT_C${}_{4}$	R_kurtosis	R_MCA_min	R_max
10	L_MPA_skew	R_LPA_std	R_LPA_NRT_C${}_{2}$	L_skew

**Table A2:** The 10 most relevant features extracted from the STANDUP2 dataset, listed according to rank for all approaches considered: lasso, random forest, concrete, and variational dropout.

Rank	Lasso	Random Forest	Concrete Dropout	Variational Dropout
1	R_MPA_skew	R_MCA_std	R_MPA_skew	R_MCA_kurtosis
2	R_MPA_max	R_MPA_kurtosis	R_LCA_kurtosis	R_MPA_skew
3	L_MCA_min	L_MCA_min	R_MCA_std	R_LCA_kurtosis
4	R_MCA_std	L_LCA_std	R_MCA_kurtosis	LPA_ETD
5	R_MCA_kurtosis	L_LCA_max	L_MCA_std	R_MPA_std
6	R_MPA_std	L_MPA_NRT_C${}_{4}$	L_LPA_min	L_LPA_HSE
7	R_max	R_LCA_min	L_MCA_min	L_LCA_std
8	L_LCA_min	L_NRT_C${}_{4}$	L_MPA_HSE	R_MPA_HSE
9	L_MPA_skew	L_LCA_min	R_LCA_min	R_MCA_min
10	R_LCA_kurtosis	L_MPA_std	R_LPA_min	R_MCA_std

**Table A3:** The 10 most relevant features extracted from the ALL dataset, listed according to rank for all approaches considered: lasso, random forest, concrete, and variational dropout.

Rank	Lasso	Random Forest	Concrete Dropout	Variational Dropout
1	L_LPA_min	R_MCA_std	R_MCA_std	R_MCA_std
2	R_MPA_max	L_LPA_min	R_MCA_kurtosis	L_MPA_skew
3	R_MCA_NRT_C${}_{5}$	L_MPA_std	L_MPA_HSE	R_MCA_skew
4	R_MCA_std	L_MPA_NRT_C${}_{3}$	R_MPA_skew	L_MPA_std
5	L_MPA_std	L_NRT_C${}_{3}$	L_MCA_skew	R_LCA_kurtosis
6	R_LCA_NRT_C${}_{9}$	R_LCA_NRT_C${}_{2}$	R_kurtosis	R_MCA_kurtosis
7	R_MPA_skew	R_MPA_kurtosis	L_LPA_min	R_MPA_std
8	R_MCA_kurtosis	R_MPA_std	L_MCA_std	LPA_ETD
9	L_LCA_NRT_C${}_{4}$	R_MCA_NRT_C${}_{2}$	R_LCA_kurtosis	R_LCA_skew
10	R_std	L_LCA_NRT_C${}_{2}$	L_MPA_skew	L_MPA_HSE
